# New NICE Guidelines on Sodium Valproate (November 2023)

**DOI:** 10.1192/bjo.2025.10582

**Published:** 2025-06-20

**Authors:** Amany Elbagoury, Asif Zia

**Affiliations:** Hertfordshire Partnership Foundation Trust, Hatfield, United Kingdom

## Abstract

**Aims:** The audit aims to ensure compliance with the new sodium valproate guidelines published in November 2023, identify areas of deviation from the guidelines, and improve patient safety and outcomes by promoting adherence to best practices.

**Methods:** Data was collected from various sources, including patients’ case notes, GP records, and attachments like acknowledgment forms. Out of 519 service users, 68 were selected based on age (55 years or younger). These were divided into two groups: 42 new cases (system alert created on 2024) and 26 current cases (system alert created 2023). After reviewing and excluding duplicates, 3 new cases and 10 current cases were analysed. Case notes and GP records were examined for risk acknowledgement forms, LFT (liver function tests), and FBC (full blood count) results. It was noted that no case records specifically mentioned these tests as part of valproate initiation or monitoring.

**Results:** New Cases: Demographics: 2 males, 1 non-binary.

Testing: LFT and FBC were conducted in 67% of cases before initiation; 100% had LFTs within six months.

Forms & Settings: All had acknowledgement forms with specialist signatures; 2 initiated in inpatient and 1 in a community setting.

Discrepancy: 67% had mismatches between alert creation and medication start dates.

Current Cases: Demographics: 8 males, 2 females.

Testing: 60% had LFT and FBC tests before initiation; only 30% had LFTs within six months.

Annual Reviews: For females, annual acknowledgement forms were completed in one of two cases; both complied with the Pregnancy Prevention Programme (PPP).

Discrepancy: All cases had alert creation mismatches.

**Conclusion:** Good Practices:

Reduced new sodium valproate cases in 2024 (from 10 in 2023 to 3).

Universal completion of acknowledgement forms for new cases.

Adherence to PPP for females and avoidance of valproate initiation in females under 55.

Areas for Improvement:

Documentation and completion of LFT/FBC tests before and after initiation.

Timely creation and removal of alerts.

Clear record of initiation dates, reasons, and follow-up plans.

Mitigation Strategies:

Training healthcare professionals on guidelines and the importance of acknowledgement forms for all patients.

Ensuring accurate alert management.

Proper documentation in discharge plans for female patients.

Verification of recent LFTs and acknowledgement forms during reviews.

